# Quantification of ferroptosis pattern in bladder carcinoma and its significance on immunotherapy

**DOI:** 10.1038/s41598-022-12712-5

**Published:** 2022-05-31

**Authors:** Xu Cheng, Yinhuai Wang, Yijian Li, Wentao Liu

**Affiliations:** Department of Urology, The Second Xiangya Hospital, Central South University, Renmin Middle Road 139, Changsha, 410011 China

**Keywords:** Cancer, Computational biology and bioinformatics, Oncology, Urology

## Abstract

The role of ferroptosis in tumor development and therapy has been previously proved. Nonetheless, its potential role in tumor microenvironment (TME) and immunotherapy for bladder carcinoma remains unclear. Based on 38 ferroptosis-related genes, the characteristic of ferroptosis patterns and interactions with immune cell-infiltrating features in 2043 bladder cancer samples were systematically investigated. We further proposed the FerrScore to quantify the ferroptosis patterns for each patient. As results, three diverse ferroptosis patterns with distinct tumor-infiltrating immune cell features were established. By determination of ferroptosis patterns of each patient, we found that high FerrScore was related to lower proportion of luminal-papillary molecular subtype, more frequent TP53 mutations, activation of immunity and stroma, and lower 5-year survival. High FerrScore also seemed to be associated with decreased neoantigen load, tumor mutational burden and poorer response to anti-PD-L1/1 therapy. External verification in two immunotherapy cohorts showed FerrScore was an independent and effective prognostic factor for therapeutic effect and survival outcome. Overall, the present study indicated the ferroptosis strongly is closely correlated with TME diversity. Evaluation of the ferroptosis patterns may strengthen the cognition of TME immune cell infiltrations and guide more individualized immunotherapeutic strategies in bladder carcinoma.

## Introduction

Ferroptosis, an iron-dependent pathway of cell death, differs from new forms of programmed cell death such as apoptosis, pyrolysis, and autophagy^1^. Several pathways such as mevalonate, iron, amino acids, NADH, phospholipids, glutathione, and iron autophagy pathways are involved ferroptosis^1–3^, and agents such as lipid peroxidation inhibitors, iron chelators, et al. are developed to inhibit the progression of ferroptosis^4^. Tumor cells, enriched in free iron and with high levels of reactive oxygen species, are more sensitive to ferroptosis^5^. Notably, ferroptosis-related genes (FRGs) such as FANCD2, GPX4, and DPP4 et al. are closely correlated to tumorigenesis and progression^6–8^, various studies have confirmed the pivotal role of ferroptosis in tumor development and therapies^9–11^. Therefore, ferroptosis can be a potential therapeutic approach to efficiently trigger cancer cell death, as alternative to the traditional treatment^12^.

Bladder cancer (BLCA) represents the tenth most common malignant tumor and the most prevalent cancer of the urinary system, with an excepted 573,278 newly diagnosed cases and 212,536 estimated deaths worldwide in 2020^13^. Although radical cystectomy followed by radiotherapy, chemotherapy and immunotherapy remains the mainstay treatment for localized BLCA, immune checkpoint inhibitors (ICIs) in treatment or neoadjuvant treatment for BLCA has been widely validated in prospective clinical trials^14–16^. However, most patients gain little or no clinical benefits from these immunotherapies^17,18^, outcomes of muscle-invasive BLCA still remain unsatisfactory^19,20^. Therapeutic response of many tumors, including BLCA, correlates with viration of tumor microenvironment (TME), the number of neoantigens, and tumor mutational burden (TMB)^21–23^. There is increasing evidence that TME is associated with the prognosis and treatment response of BLCA and other types of cancer^24–26^.

Recently, Wang et al. reported that CD8 + T cells promote tumor ferroptosis during cancer immunotherapy treatment^27^. Additionally, ferroptosis seems more immunogenic than apoptosis due to release of damage-associated molecular patterns family, which in turn exacerbates inflammatory reactions^28^. Moreover, Liu et al. have developed the ferroptosis potential index (FPI) to reveal the relationship between ferroptosis and the cancer hallmarks, tumor microenvironment, drug resistance, and patients’ survival^29^. These findings suggest that the ferroptosis plays an essential role in the tumor microenvironment (TME). However, literatures focus on the role of ferroptosis in immunotherapy for BLCA is limited, and most of those scattered studies focused on the link between ferroptosis and individual immune cell or functions, a comprehensive analysis of the relationship between ferroptosis and TME in BLCA will be helpful.

Herein, we integrated the genomic information of 2043 BLCA samples to comprehensively evaluate the ferroptosis patterns, and correlated the ferroptosis pattern with the TME cell-infiltrating characteristics. We revealed three distinct ferroptosis patterns, and surprisingly found that the TME cell-infiltrating characteristics and prognosis under these three patterns were significantly different. Thus, we first established a set of scoring system to quantify the ferroptosis patterns for each BLCA patient based on the mRNA expression profiles of FRGs. The scoring system may assist oncologists to make more efficient and individualized immunotherapeutic strategies.

## Method

### BLCA dataset collection and preprocessing

We retrospectively retrieved the BLCA RNA sequencing (RNA-seq) data and matched clinical features from the Carcinoma Genome Atlas (TCGA), Gene-Expression Omnibus (GEO) and ArrayExpress databases. Patients lacked survival information were excluded and totally 9 eligible BLCA datasets (GSE13507, GSE31684, GSE32548, GSE48075, GSE48276, GSE70691, TCGA-BLCA, E-MTAB-4321 and IMvigor210) were included in the subsequent investigation. In Affymetrix platform, the raw “CEL” file was performed with background adjustment and normalization by using the ‘affy’ and ‘simpleaffy’ packages while other platforms were directly downloaded a normalized matrix file. Besides, the RNA sequencing data (FPKM) from TCGA database was transferred to transcript per kilobase million (TPM) by using the TCGAbiolinks package. Subsequently, we made an integration of these data into a meta-dataset for removing batch based on ComBat function within sva package^30^. Table S1 presented the baseline of 9 eligible datasets of BLCA. Additionally, we carried out Copy Number Variation (CNV) analysis on the base of downloaded TCGA dataset which provided somatic mutation data.

### Unsupervised clustering for ferroptosis-related genes

Firstly, we performed the unsupervised clustering analysis to identify different ferroptosis forms based on the expression of 38 ferroptosis related genes (FRGs) from 6 GEO datasets. What’s more, we acquired these mentioned ferroptosis genes from GSEA databases. In addition, consensus clustering algorithmic was applied for determining cluster amount and stability. Notably, the mentioned steps were implemented with the ConsensuClusterPlus package and 1000 times repetitions were conducted to ensure the stability of classification.

### Gene set variation analysis and functional annotations

To further evaluate the biology distinction in autophagy subdivided types, we performed the Gene Set Variation Analysis (GSVA) analysis by “GSVA” packages^31^, and the “c2.cp.kegg. v6.2. symbols” gene set was chosen as the reference. Statistical significance was set as adjusted *p* value < 0.05^32^.

### Estimation of TME cell infiltration

We estimated the immune cell infiltration in the tumor microenvironment (TME) by single-sample GSEA. Based on Charoentong’s study, we obtained the data of these immune cell types gene set, and the immune infiltration scores were defined as the mean of standardized values of immune cells including CD8^+^ T cells, CD4^+^ T cells, immature B cells, memory B cells, etc.^33^. Moreover, we assessed the enrichment scores of immune cells and function of BLCA specimens, and Kruskal–Wallis test was used for the comparison.

### Recognition of differentially expressed genes among diverse ferroptosis phenotypes

According to the mRNA expression profiles of 38 FRGs, patients were classified as three ferroptosis phenotypes. Moreover, we identified differentially expressed genes (DEGs) in different ferroptosis phenotypes based on the empirical Bayesian approach using the package “limma”^34^. The adjusted value of *p* < 0.001 was set as threshold.

### DEGs clustering and ferroptosis score

We developed a ferroptosis score to quantify the ferroptosis form in a BLCA patient. The steps to establish the signature of ferroptosis genes were presented as below: first, we selected the overlapping DEGs determined by ferroptosis clustering and normalized among GEO dataset. Next, patients were separated into groups to conduct the further unsupervised clustering analysis of overlapping DEGs. Then, we defined the gene cluster number and its stability based on the consensus clustering algorithmic rule. Subsequently, we performed the univariate Cox regression analysis to investigate the prognostic significance of the respective gene in the TCGA-BLCA dataset. Genes with significant prognosis were used for further study (*p* < 0.05). Finally, the above genes were fitted into LASSO Cox regression study to reduce the dimensionality and then select representative decision makers. The score of ferroptosis was generated by the following formula:

$$ {\text{FerrS}}core = \sum\limits_{i = 1}^{n} {Coefi*Expri} $$

where Expri presents the expression level of ferroptosis-related signature genes and Coefi stands for LASSO Cox regression coefficient.

### Correlation between signature of ferroptosis genes and other relevant biology processes

Mariathasan et al. used gene sets to classify genes related to different biological processes, including (1) DNA replication; (2) Cell cycle; (3) Fanconi anemia; (4) DNA damage repair; (5) Homologous recombination; (6) Nucleotide excision repair; (7) Immunization checkpoint; (8) Fibroblast Growth Factor Receptor 3 (FGFR3)-related genes; (9) Antigen processing machinery; (10) CD8^+^ T effector; (11) epithelial-mesenchymal transition (EMT) marker covering EMT3, EMT2 and EMT1; (12) Angiogenesis; (13) pan-fibroblast transforming growth factor (TGF)-β response signature (Pan-F-TBRS)^35^. Moreover, we conducted a correlation analysis to estimate the relationship between signature of ferroptosis genes and other relevant biology processes.

### Data collection and assessment of response to immune checkpoint blockade (ICB)

Expression profiles of immune checkpoint blockade genes with complete clinical information were systematically searched from public data. We included one anti-PD-L1 and one anti-PD-1 immunotherapy datasets to calculate the FerrScore, as follows: 1) the advanced urothelial carcinoma treated by atezolizumab, antibody for PD-L1 (IMvigor210 dataset, by complying with the Creative Commons 3.0 License, 30; 2) the metastatic melanoma with intervention of pembrolizumab, antibody for PD-1 (GSE78220 dataset originating in GEO)^22^. The DEseq2 R package and limma R package were utilized to normalize the raw data. Furthermore, transformation from the count value into the TPM value was performed, respectively.

### Clinical samples

We retrospectively collected the clinical data of patients with bladder cancer receiving anti-PD-1 therapy (01.2021–04.2021), and retrieved the cancer samples in our biobank for qRT-PCT test. The inclusion criteria of this study were as follows: (1) age 18–75 years; (2) patients who were pathologically diagnosed with bladder urothelial carcinoma (T2-4aN0M0); (3) patients scheduled for radical cystectomy; (4) combined positive score (CPS) of PD-L1 ≥ 10%. ORR was defined as the percentage of participants in the analysis population witha Complete Response (CR: disappearance of all target lesions) or a Partial Response (PR: at least a 30% decrease in the sum of diameters of target lesions) per RECIST 1.1. ORR was assessed by BICR in participants with strongly PD-L1 positive tumors (CPS ≥ 10%). This study was approved by the Ethics Committee of the Second Xiangya Hospital of Central South University, and informed consent was obtained from all included participants.

### Statistical analysis

The study endpoint in above different datasets is all overall survival, which is defined as the period span from the initial treatment to the all-cause death. The correlation between the TME infiltrating immunization cells and expression of ferroptosis genes was calculated by spearman and distance correlation analysis. One-way ANOVA and Kruskal–Wallis tests were used for comparisons among three or more groups. The best cut-off point of FerrScore in every separated group was identified by the survminer package to assess the correlation between FerrScore and patients' survival. To control the calculated batch effect, we repeatedly tested the cut-off values repeated to determine the statistical value of maximal rank, and then classified the individuals into FerrScore-high and the FerrScore-low patients by the statistics. Survival analyses of subgroups for overall survival (OS) and progression-free survival (PFS) were performed using Kaplan–Meier estimates, log-rank testing, and Cox regression modeling. The FerrScore was validated by the receiver-operating characteristic (ROC) curves. Besides, we performed multivariate prognostic analysis for FerrScore in E-MTAB-4321 and TCGA dataset, and the results were visualized by R package- ‘forestplot’. Moreover, the mutation landscape of high and low FerrScore individuals from TCGA dataset was presented by the maftools package. We illustrated the CNV landscape of 38 FRGs that located in human chromosomes using the RCircos package. Two-sided *p* < 0.05 was considered statistically significant. All statistical investigation was conducted using R 4.0.5 software.

### Ethics approval and consent to participate

The study was conducted in accordance with the Declaration of Helsinki (as revised in 2013). The study was approved by the ethics committees at the Second Xiangya Hospital of Central South University. The patient data in this work were acquired from the publicly available datasets whose informed consent of patients was complete.

## Results

### Three ferroptosis patterns mediated by 38 ferroptosis-related genes

**Figure 1:**
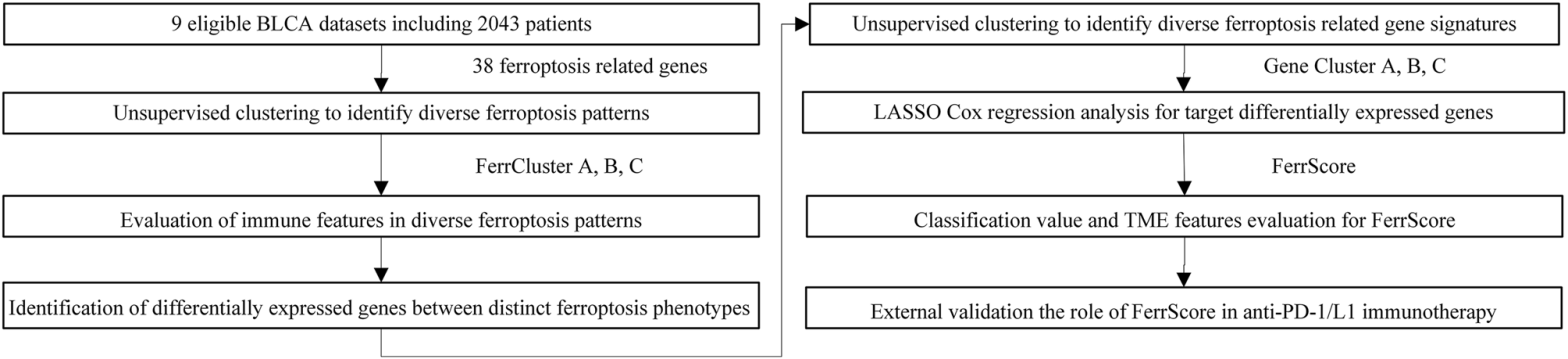
The flow diagram of our main analysis.

**Figure 2:**
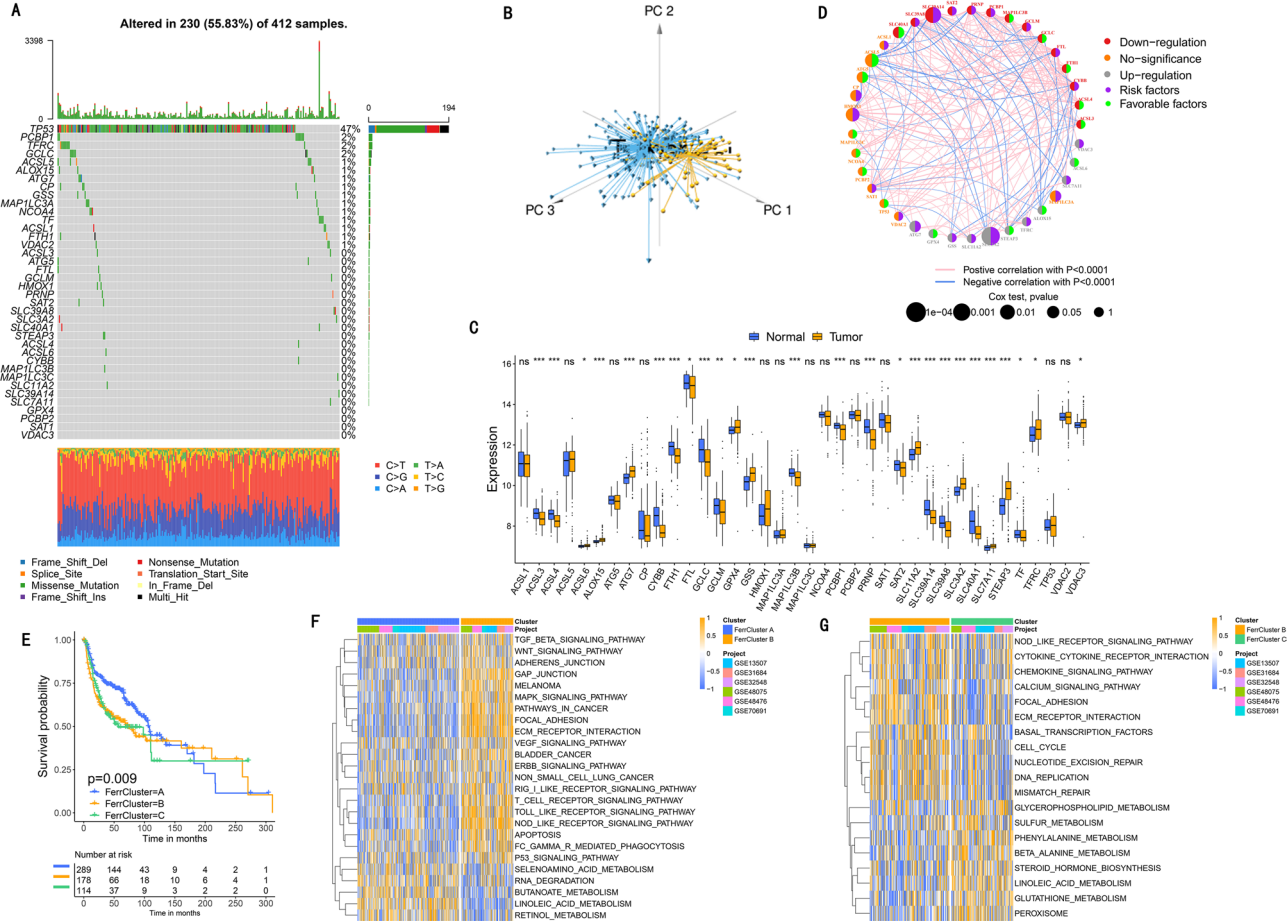
The genetic and expression variation landscape of ferroptosis-related genes related to bladder cancer. (**A**) The mutation frequency of 38 ferroptosis-related genes in 412 patients who have bladder cancer from TCGA dataset. Each patient was shown in individual column, among which TMB was shown in the bar graph on top, with the mutation frequency of each regulator shown as number on the right, the ratio of each variant type was shown in the bar graph on the right, and conversion rate of each sample was shown in the stacked bar chart below. (**B**) Analysis performed for principal component of 38 ferroptosis-related genes' expression profiles to tell apart tumors and normal samples in TCGA dataset. Tumors were marked in blue and normal samples in yellow (**C**) The transcripts of 38 ferroptosis-related genes on control samples and bladder cancer. Yellow shows tumor and blue represents the normal. The interquartile range of values was represented by the boxes' upper and lower ends, the median value is represented by the lines in the boxes, and outliers were shown by dots in black. (**D**) Interaction between ferroptosis-related genes in bladder cancer. The effect of each gene on the prognosis is indicated by the size of circle, and by Log-rank test. the scales of *P* value were less than 0.0001, 0.0001, 0.01, 0.05 and 0.1. In the circle, purple dots indicated risk factors of prognosis while green dots indicated protective factors of prognosis. The interaction between genes were shown by linking lines and the correlation strength between genes were shown by thickness. Blue indicates negative correlation and red indicates positive correlation. (**E**) Analysis supported by Kaplan–Meier curves of survival for these ferroptosis subtypes regarding 481 patients who have bladder cancer from six GEO datasets (GSE13507, GSE31684, GSE32548, GSE48075, GSE48476, GSE70691), including 289 patients in Ferrcluster-A, 178 patients in Ferrcluster-B, and 114 patients in Ferrcluster-C. A great difference of the survival of these ferroptosis subtypes was shown by the Kaplan–Meier curves, along with Log-rank *p* value, which is 0.009. Compared to other two Ferrclusters, Ferrcluster-A is significantly better on survival. (**F**–**G)** Analysis of GSVA enrichment, which suggested the status of biological pathways' activation in different ferroptosis subtypes. These biological processes were visualized by heatmap, with activated pathway shown in brown and inhibited pathway shown in blue. For sample annotations, it used the bladder cancer datasets. (**F**) Ferrcluster-A versus Ferrcluster-B, and (**G**) Ferrcluster-B versus Ferrcluster-C. The value of *p* is graphically shown by asterisks, of which * means that *p* is less than 0.05, ** means less than 0.01 and *** means less than 0.001.

An overview of the study design was shown in Fig. 1. 38 FRGs were finally retrieved from the GSEA database. The genetic and expression variation landscape of FRGs varied a lot in different BLCA samples (Fig. 2A–C and S1A,B). In addition, the 38 FRGs' expression profiles can very well distinguish tumor from normal sample (Fig. 2B,C). Six GEO datasets (GSE13507, GSE31684, GSE32548, GSE48075, GSE48476, GSE70691, Table S1) were included in a Meta dataset. The 38 FRGs interacted with each other and had prognostic value for BLCA (Fig. 2D and S1C). According to expression level of 38 FRGs, three diverse patterns with distinct survival were recognized, named FerrCluster A-C (Fig. 2E and S2A).

### Immune features in diverse ferroptosis patterns

**Figure 3:**
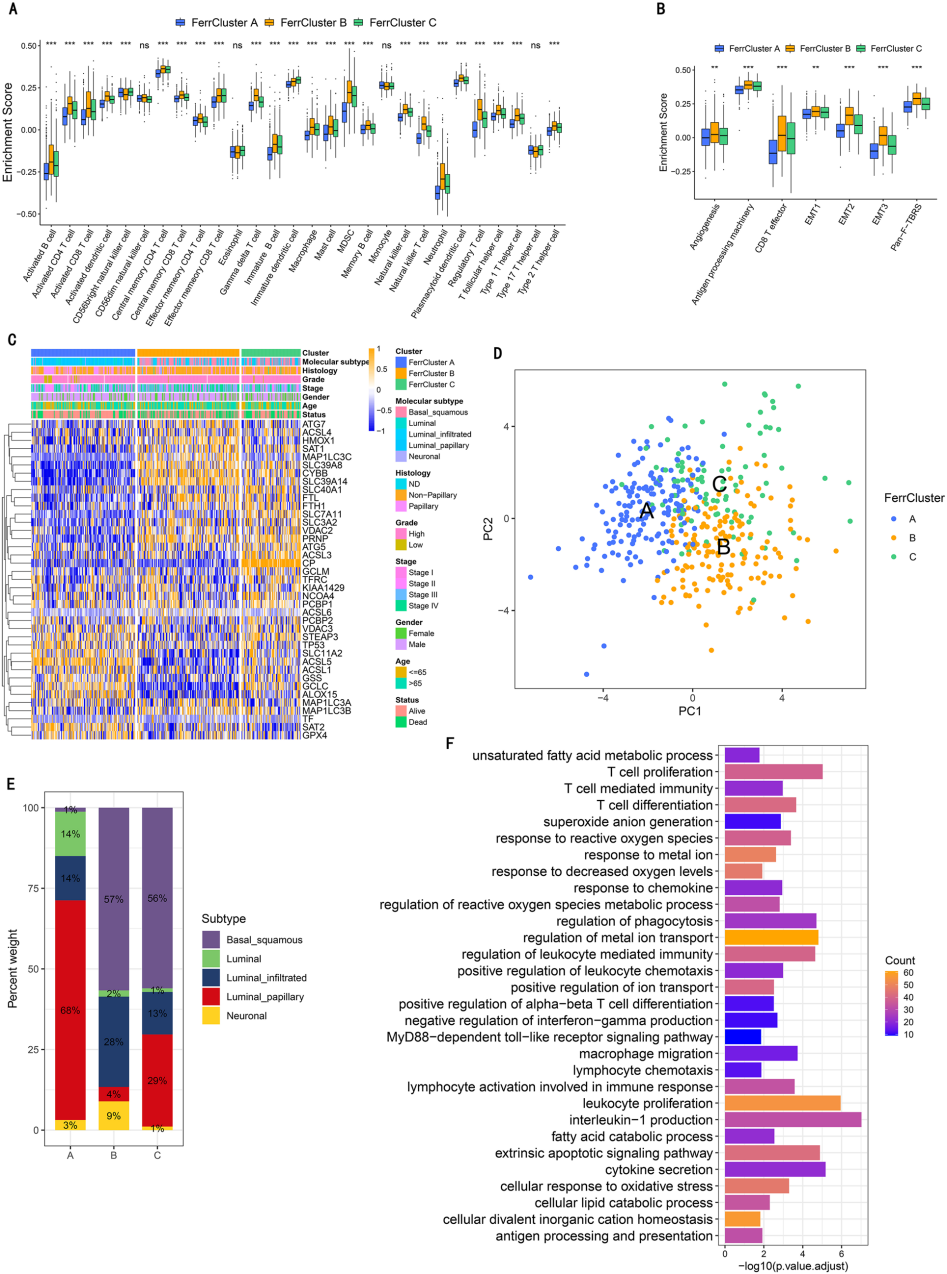
Features of TME transcriptome property and cell infiltration in different ferroptosis forms. (**A**) The abundance of individual TME infiltrating cell in these ferroptosis forms. Their interquartile value ranges were indicated by lower and upper ends of boxes. The median value was indicated by those lines in the boxes, and outliers were shown with dots of black color. The value of *p* is graphically shown by asterisks, of which * means that *p* is less than 0.05, ** means less than 0.01 and *** means less than 0.001. (**B**) Diversities in stroma-activated pathways, such as angiogenesis, TGF beta and EMT pathways among these ferroptosis forms. The test called one-way ANOVA was applied to check group diversity among these ferroptosis forms statistically. The value of *p* is graphically shown by asterisks, of which * means that *p* is less than 0.05, ** means less than 0.01 and *** means less than 0.001. (**C**) Non-monitored clustering of 38 ferroptosis-related genes in the TCGA: BLCA dataset. Annotations for selected patient including the ferroptosis, TCGA molecular subtypes, histology, stage of tumor, grade, status of survival and age. High gene expressions were marked in brown and low gene expressions were marked in blue. (**D**) Analysis of principal component for the transcriptional feature of these ferroptosis forms, indicating a significant differential gene expression among different subtypes. (**E**) The distribution of TCGA molecular subtypes in these ferroptosis forms. Basal_squamous subtype, purple; Luminal subtype, green; Luminal_infiltrated subtype, blue; Luminal_papillary subtype, red; and Neuronal subtypeb was shown in yellow. (**F**) Functional annotation for genes relevant to ferroptosis according to GO enrichment analysis. The number of enriched genes were indicated by the bar graph's color depth.

The transcriptome heat map describes the most abundant differentially expressed genes (DEGs) with different biological functions in the three clusters (Fig. 2F–G). Surprisingly, FerrCluster-B was noticeably rich in both innate and activated immune cell infiltration (Fig. 3A), which was related to stromal activation, such as angiogenesis pathways, pan-fibroblast TGF-β response and EMT (Fig. 3B). Therefore, we hypothesized that stromal activation in FerrCluster-B inhibited the antitumor effect of immune cells, leading to poor survival in FerrCluster B (Fig. 2E). Of note, FRG expression did not alter tumor's TME-infiltrating cell types (Fig. S2B).

Besides, correlation between each TME-infiltrated cell and the FRG was shown in Fig. S3A. FRG CYBB, a major component of the phagocytic oxidase system, was up-regulated with higher immune-score, suggesting its role in immune cell infiltration (Fig. S3B–D). Further pathway enrichment analysis revealed that up-regulated CYBB would enhance different pathways to activate immunity (Fig. S3E), which accompanied with up-regulated PD-L1 and PD-1 (Fig. S3D–E). In GSE78220, high CYBB expression was related to better survival in patients with anti-PD-1 immunotherapy (Fig. S3F). However, this effect was not observed in IMvigor210 (Fig. S3G). Nevertheless, we speculated that CYBB-mediated ferroptosis might induce immune cell activation, which enhances the anti-tumor immune responses in tumors.

To further explore ferroptosis phenotypes in the BLCA dataset in TCGA, we performed cluster analysis to generate three distinct ferroptosis phenotypes (Fig. 3C,D and S4A–D), with different expression levels of FRG. FerrCluster B was obviously related to stromal activation, tumor progression, basal_squamous subtype, worse histological grade and survival (Fig. 3C,E and S4E,F).

### Ferroptosis pattern-related gene signatures and their functional annotation

**Figure 4:**
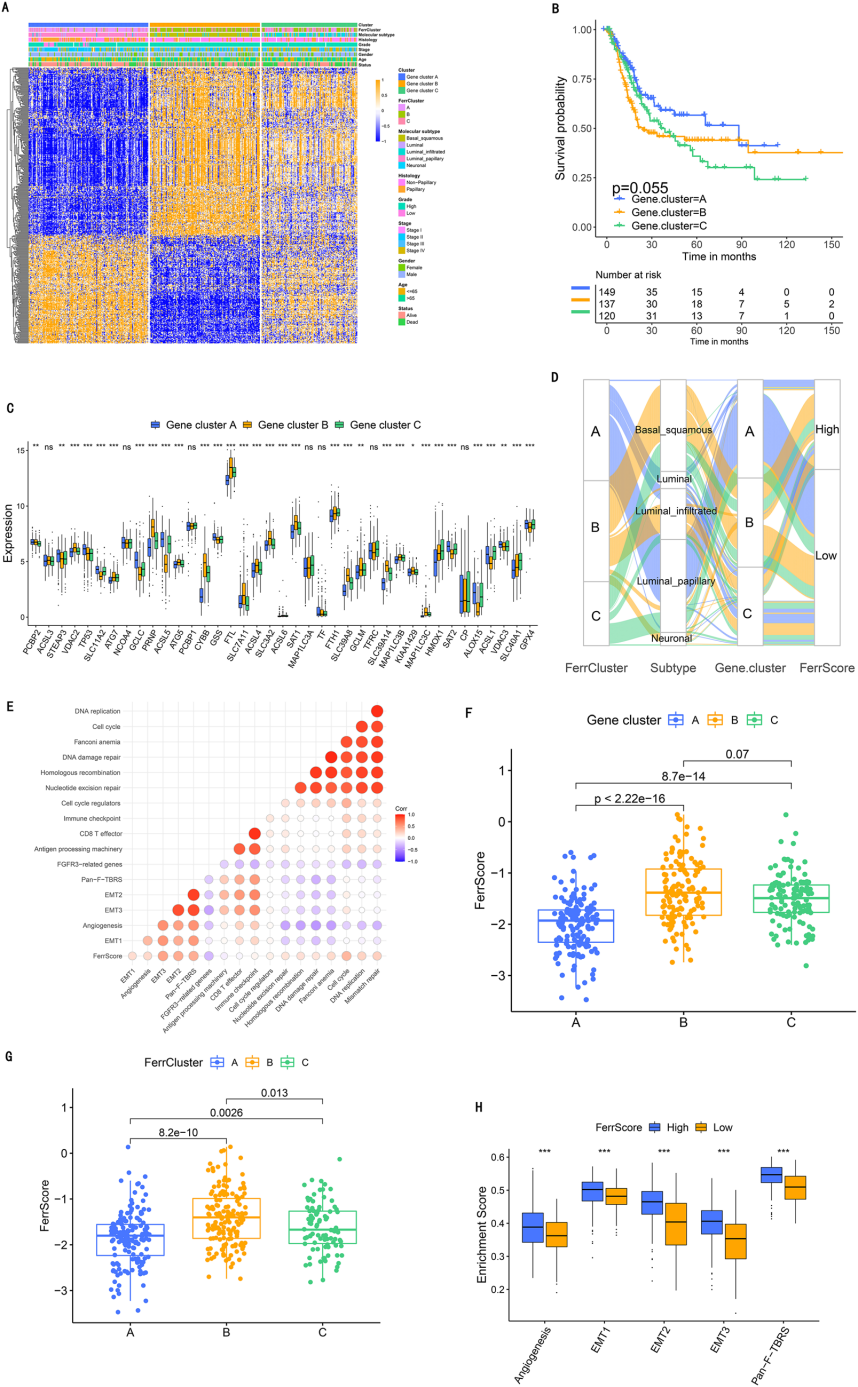
Signature construction of ferroptosis genes. (**A**) Unmonitored clustering of overlapping ferroptosis subtype-related genes in TCGA datasets to attribute patients to different genomic subtypes, named as ferroptosis gene cluster A, B and C. Patient annotations were utilized by the gene clusters, ferroptosis subtypes, TCGA molecular patterns, stage of tumor, grade, histology, status of survival, gender and age. (**B**) Kaplan–Meier curves suggested that ferroptosis genomic phenotypes were significantly relevant to the 406 patients' survival in TCGA: BLCA dataset, among the cohort, 149 patients were classified to cluster A, 137 patients were classified to cluster B and 120 patients were classified to cluster C (*p* = 0.055, Log-rank test). (**C**) The results of 38 ferroptosis-related genes in these gene clusters. The interquartile range of values was represented by the boxes' upper and lower ends, the median value is represented by the lines in the boxes, and outliers were shown by dots in black. The value of *p* is graphically shown by asterisks, of which * means that *p* is less than 0.05, ** means less than 0.01 and *** means less than 0.001. The test called one-way ANOVA was applied to check group diversity among these ferroptosis forms statistically. (**D**) The changes of ferroptosis clusters, TCGA molecular patterns, ferroptosis gene cluster and FerrScore shown by alluvial diagram. (**E**) Spearman analysis showing the connection between FerrScore and the known gene features in TCGA: BLCA dataset. Blue color represented negative correlation and red color represented positive correlation. (**F**) Diversities of FerrScore in these gene clusters. The difference of the gene clusters was compared statistically with the Kruskal–Wallis test (*p* less than 0.05). (**G**) Diversities of FerrScore in these ferroptosis subtypes in TCGA: BLCA dataset (*p* less than 0.05, Kruskal–Wallis test). (**H**) Diversities of stroma-activated pathways in groups of high and low FerrScore. EMT, epithelial-mesenchymal transition; Pan-F-TBRS, pan-fibroblast transforming growth factor (TGF)-β response signature. The interquartile value ranges were indicated by boxes lower and upper ends. The median value was indicated by those lines in the boxes, and outliers were shown with dots of black color. The value of *p* was graphically shown by asterisks, (***:*p* less than 0.001).

Then, we identified 456 ferroptosis pattern-related DEGs which were co-expression in the three phenotypes (Fig. S4G). GO enrichment analysis showed these genes were significantly enriched in ferroptosis and immunity (Fig. 3F). The further cluster analysis of 456 genes divided patients into three clusters named as ferroptosis gene cluster A-C (Fig. S5A–D and 4A). The high grade, advanced stage, basal_squamous type and worse survival were related to ferroptosis gene cluster B (Fig. 4A,B). The expression of 38 FRGs varied in the three gene clusters (Fig. 4C).

To further explore the role of distinct ferroptosis phenotype in the TME immune regulation, we searched the published literature to extract target cytokines and chemokines, which were related to immune activation, immune checkpoints and (TGF)-β /EMT pathway, respectively^30,31^. (TGF)-β /EMT pathway was up-regulated in ferroptosis gene cluster B, indicating this cluster was stroma-activated and cancer promotion (Fig. S5E–H). These findings uncovered the important role ferroptosis played in generation of distinct clinical, transcriptome traits and TME features.

### Development of FerrScore and its prognostic value

**Figure 5:**
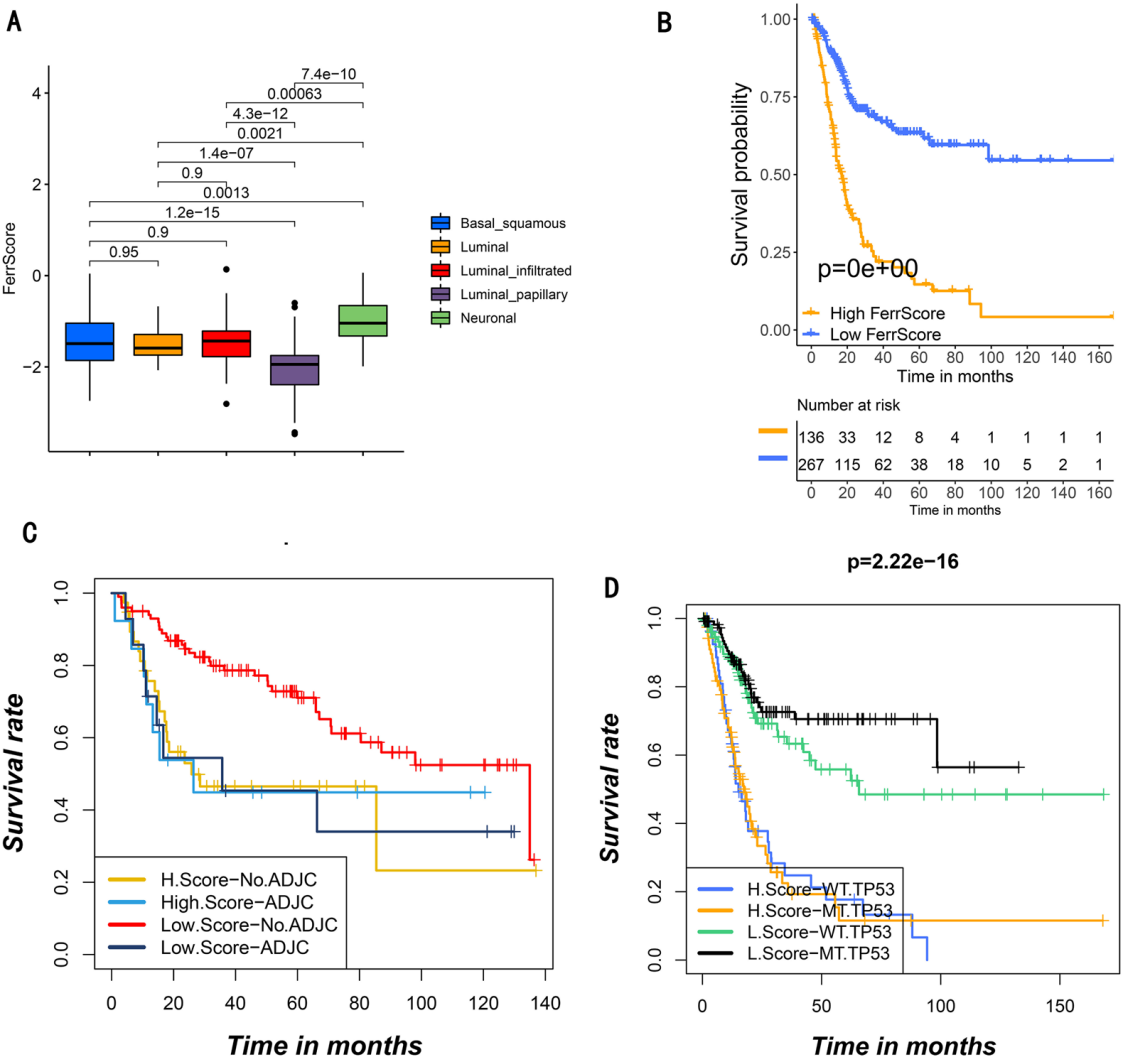
Features of ferroptosis in different TCGA molecular patterns and somatic mutation of tumor. (**A**) Diversities in FerrScore between these TCGA molecular patterns. The difference between five TCGA molecular patterns was calculated statistically by the Kruskal–Wallis test (*p* value less than 0.05). (**B**) Survival analysis performed with Kaplan–Meier curves in low (267 patients) and high (136 patients) FerrScore patients in TCGA: BLCA dataset (*p* less than 0.05, Log-rank test). (**C**) Survival analysis performed with Kaplan–Meier curves in high and low FerrScore patients receiving adjuvant chemotherapy or not. ADJC means adjuvant chemotherapy; H means high and L means low (*p* = 0.006, Log-rank test). (**D**) Analysis supported by Kaplan–Meier curves for subgroup patients formed by both FerrScore and TP53 mutation, listing as high: mutant type: MT, wild type: WT, high: H, low: L (*p* less than 0.05, Log-rank test).

We further developed a scoring system based on the ferroptosis phenotype-related DEGs to quantify the ferroptosis pattern of individual patients, and termed it as FerrScore (Fig. 4D), which was correlated with well-known signatures (Fig. 4E). We found a higher FerrScore in ferroptosis gene cluster B was associated with stromal activation (Fig. 4F–H), and the luminal_papillary type was also related to low FerrScore (Fig. 5A).

Next, patients were divided into low or high FerrScore group by the cutoff value -1.42. Patients with low FerrScore obtained better survival (*p* < 0.05; Fig. 5B), and FerrScore can be an independent prognostic factor for this (HR 4.30(2.97 − 6.22)) (Fig. S6A). Among patients without adjuvant chemotherapy, low FerrScore was related to more therapeutic advantages (Fig. 5C, *p* < 0.05), which was not interfered by TP53 mutation (Fig. 5D). Furthermore, younger, male, low grade and early stage were significantly related to a lower FerrScore, with better clinical outcomes (Fig. S6B). The mentioned results demonstrated FerrScore could be also used to evaluate individual characteristics.

### External verification and the role of ferroptosis pattern in ICB therapy

FerrScore signature built in TCGA: BLCA dataset was validated in other independent BLCA datasets for the relevant prognostic value (GSE13507, *p* < 0.05; GSE31684, *p* < 0.05; GSE32548, *p* < 0.05; GSE48075, *p* < 0.05; GSE48276, *p* < 0.05; Combined GEO datasets, *p* < 0.05. Fig. S7A–F). ROC curves showed a better prediction accuracy of FerrScore in 3 and 5-years (Fig. S7G,H).

**Figure 6:**
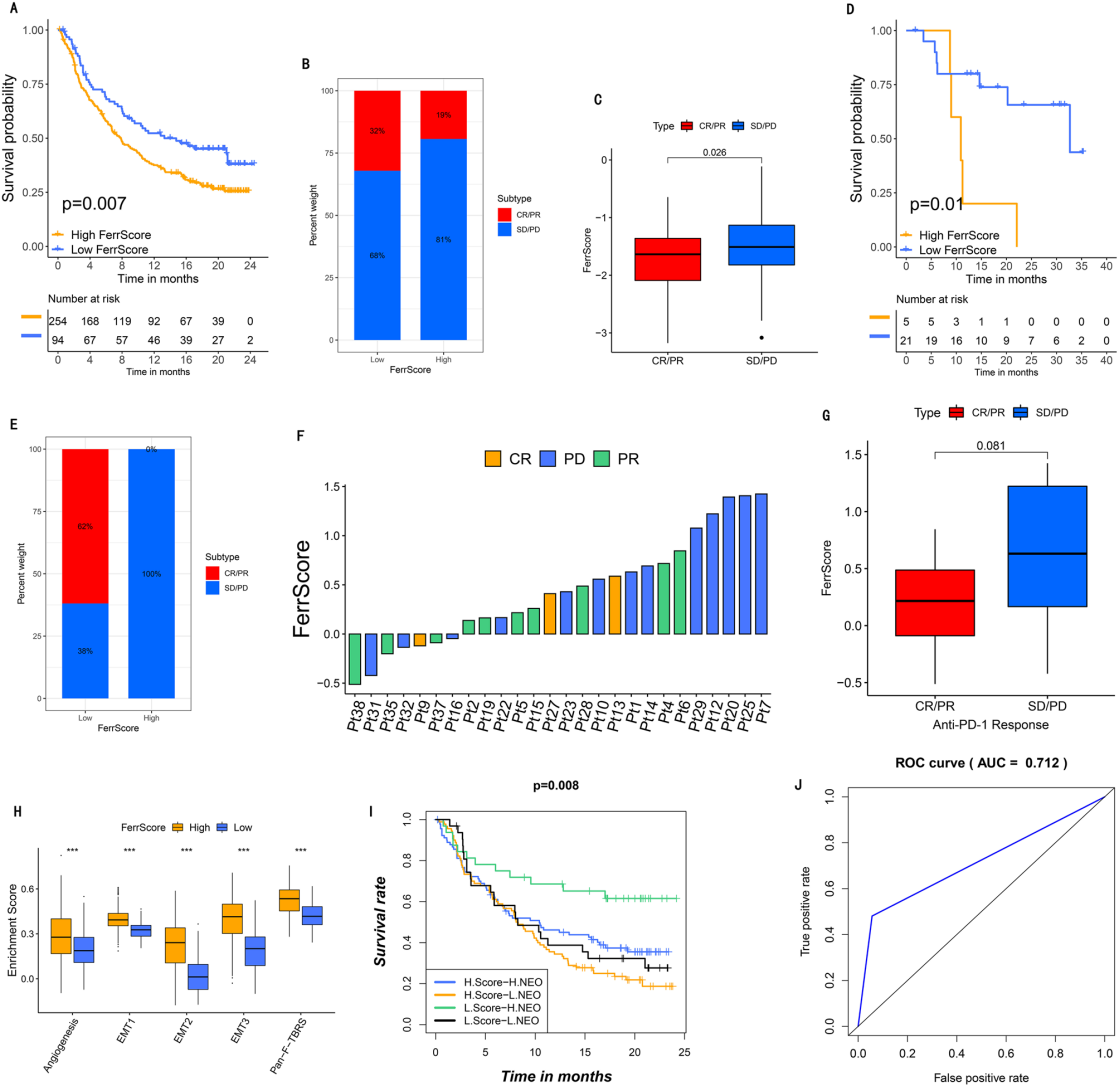
The role ferroptosis subtypes playing in anti-PD-L1/1 immunotherapy. (**A**) Survival analysis performed with Kaplan–Meier curves between low (94 patients) and high (254 patients) FerrScore groups in the anti-PD-L1 immunotherapy dataset (IMvigor210 dataset; *p* = 0.004, Log-rank test). (**B**) The effective rate of the high or low FerrScore patients receiving PD-L1 blockade immunotherapy. SD means stable disease; PD means progressive disease; CR means complete response; and PS means partial response. (**C**) FerrScore in two distinct anti-PD-L1 immunotherapy outcome groups. (**D**) Survival analysis performed with Kaplan–Meier curves between low and high FerrScore patient receiving anti-PD1 immunotherapy (GSE78220 dataset; *p* = 0.01, Log-rank test). (**E**) The effective ratio in low and high FerrScore patients receiving PD-1 blockade immunotherapy. (**F**) Correlative analysis between FerrScore and treatment effect of anti-PD-1 immunotherapy. Pt means patients, PD was shown in blue color, PR was shown in green color and CR was shown in yellow color. (**G**) FerrScore in two different anti-PD-1 immunotherapy outcome groups. (**H**) Diversities in stroma activated pathways between low and high FerrScore groups in patients receiving anti-PD-L1 immunotherapy (***p less than 0.001). (**I**) Survival analysis performed with Kaplan–Meier curves for in anti-PD-L1 immunotherapy dataset classified with both neoantigen burden and FerrScore. H: high; L: low; NEO: neoantigen burden (*p* = 0.008, Log-rank test). (**J**) The predict value of FerrScore in patients receiving anti-PD-L1/1 treatment (AUC, 0.712).

Patients with low FerrScore gained obviously better tumor control effect and survival outcomes in the two datasets (IMvigor210: Fig. 6A–C, *p* < 0.05; GSE78220: Fig. 6D–G, *p* < 0.05). Low FerrScore was related to therapeutic advantages and clinical response to ICB therapy (Fig. 6B–C and E–G). The stromal activation was observed in the high FerrScore tumors, thereby mediating the immunization tolerance of the tumors (Fig. 6H). High tumor neoantigen load, which was closely related to immunotherapy, and low FerrScore predicted a better survival (Fig. 6I). These findings suggest that the quantification of ferroptosis patterns is an effective biological marker for prognosis clinical responses to immunotherapy (Fig. 6J).

### Validation of FerrScore in clinical samples

**Figure 7:**
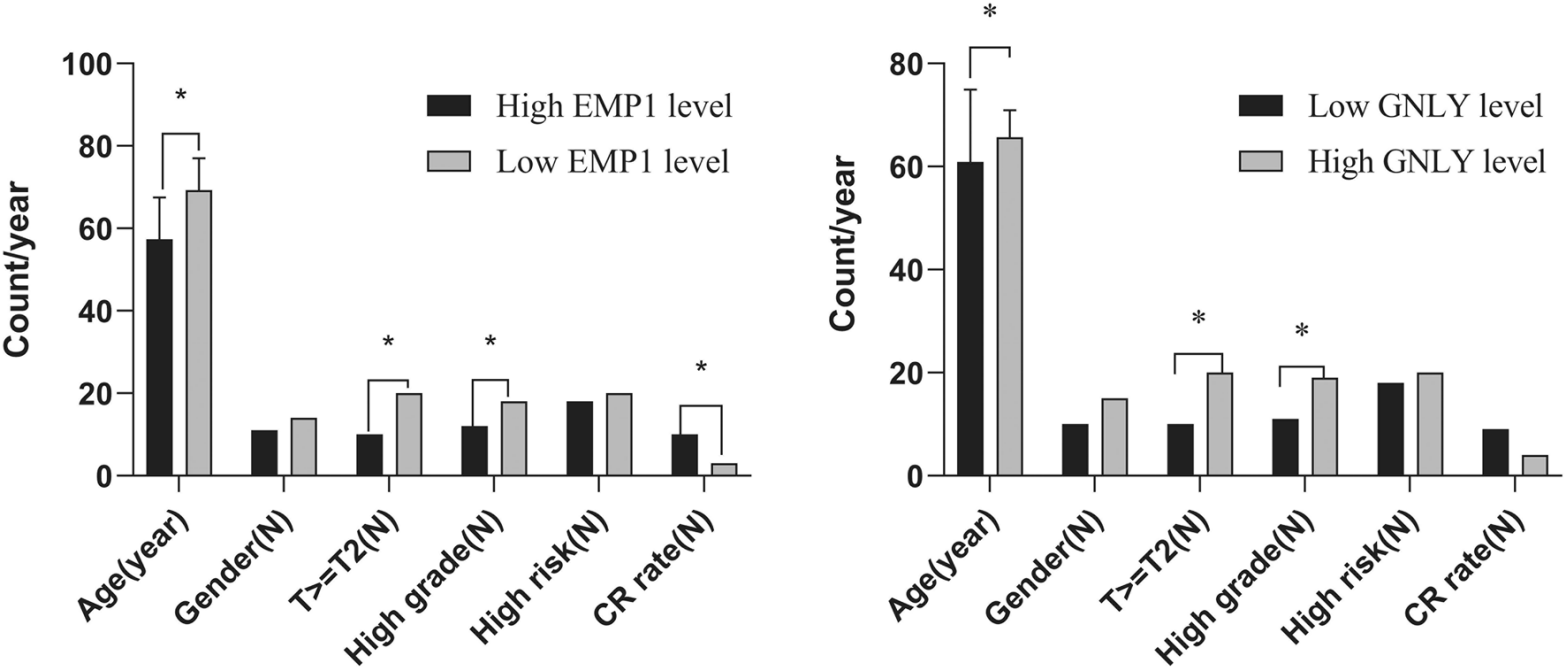
Validation of selected genes of FerroScore in clinical samples. High EMP1 level, contributing to high FerrScore was associated with advanced T stage, high grade tumor and lower CR rate. While, low GNLY level, contributing to high FerrScore was associated with more aged patients, advanced T stage, high grade tumor and lower OR rate. (N: couts for male gender, T > T2, high grade, high risk and omplete response patients (CR)).

In the present study, FerrScore was established on the coefficients of the following 18 ferroptosis related genes (Supplementary materials). Because FerrScore is established by the expression level of multiple genes, it is difficult to detect the expression of each gene. Among these genes, EMP1 and GNLY were the most significant factors with opposite effect according to their coefficients, so we chose the two genes to validate FerrScore. Finally, 40 patients with ORR data were enrolled in the study, including 20 patients with high EMP1 expression, 20 patients with low EMP1 expression, 20 patients with high GNLY expression and 20 patients with low GNLY expression, respectively. The results were shown in the Fig. 7. High EMP1 level, contributing to high FerrScore, was associated with advanced T stage, high grade tumor and lower CR rate. While, low GNLY levelcontributing to high FerrScore, which was associated with more aged patients, advanced T stage, high grade tumor and lower OR rate. The above results were consistent with our previous data in Fig. S6, so we could make a conclusion that additional validation still support the established FerrScore.

## Discussion

Ferroptosis, a newly discovered modality of programmed cell death, was found correlated with tumor drug resistance, tumor immune environment and cancer progression, and its role in BLCA had attracted attention^36^. Therefore, the clinical features and TME features exhibited by BLCA with different ferroptosis patterns were comprehensively analyzed.

We classified three ferroptosis patterns with different TME features. According to the results, FerrCluster B showed the most adaptive immunity, innate immunity and stroma activation, among which, stroma activation was considered T-cell suppressive. Therefore, after fully exploring the TME cell infiltration induced by diverse ferroptosis patterns, it was no wonder FerrCluster B had an activated innate immunity but a poor prognosis. Then, three ferroptosis gene clusters with different clinical outcomes and immune cell infiltration were generated among DEGs related to FRGs features. Similar to the clustering of ferroptosis pattern, ferroptosis gene cluster B was also correlated with activated innate immunity and stroma but a poor prognosis. Further, based on ferroptosis phenotype-related DEGs, a scoring system named FerrScore was developed and validated as an independent risk factor of prognosis for patients with BLCA. Thus, the correspondence among ferroptosis pattern- ferroptosis gene cluster-FerrScore was uncovered according to our results.

Considering the close relationship between TME cells and FerrScore, we made further exploration and found that FerrScore can also accurately predict immunotherapy response, which implied FerrScore as promising tool to select patients sensitive to immunotherapy^35^. Cancer, driven by exposure to excess iron, is a genetic disease caused by the accumulation of somatic cell mutations^37^. The present results also indicated a significantly negative correlation between FerrScore and tumor mutational burden (Fig. S8). Studies revealed that high tumor mutational burden independently attain the highest response rate to cisplatin-based chemotherapy and checkpoint inhibitors for patients with BLCA, which consistent with our results^38^. FerrScore could be independent of tumor mutational burden in predicting the responsiveness to immunotherapy.

In this study, we identified 456 ferroptosis phenotype-related DEGs, which were markedly enriched in ferroptosis and immunity. Although the iron therapeutics in cancer has been proposed recently, it has not yet achieved satisfactory clinical use owing to modest results from clinical trials or laboratory-only results^39^. It is the process upon iron addicted to ferroptosis resistance that abnormal proliferation of tumor cells requires large amounts of iron to maintain DNA synthesis and repair as well as cellular energy^40^. According to our data, FRG CYBB mediated ferroptosis was speculated to promote the activation of TME DCs, thus enhancing the intra-tumoral immunological antitumor response in BLCA. CYBB may be a potential therapeutic target, which should be further validated by experiments.

In conclusion, the comprehensive, integrated analysis of three ferroptosis patterns uncovered an extensive regulatory mechanism by which they affect TME and TME-related BLCA prognosis. The developed FerrScore can interpret diverse clinical features affected by ferroptosis and discriminate their clinical use in immunotherapy, which might guide urologist to more accurate immunotherapy strategies in BLCA. However, further experimental validation and prospective clinical studies are still required.

## Supplementary Information

Supplementary Information.

## Data Availability

Publicly available datasets and online tools were utilized in this study. These resources could be found in main text.
