# Supplementary material for: Quantification of ferroptosis pattern in bladder carcinoma and its significance on immunotherapy
Source: Sci Rep. 2022 May 31;12:9066. doi: 10.1038/s41598-022-12712-5 (PMC9156752; doi:10.1038/s41598-022-12712-5)
Supplement: Supplementary file 1 — Supplementary Information. [file 41598_2022_12712_MOESM1_ESM.pdf]

Table S1: The gene expression profiles of bladder cancer included in this study

Accession number	Source	Number of patients	Survival
TCGA: BLCA	Illumina RNAseq	432	OS
GEO: GSE13507	Illumina human-6 v2.0 expression beadchip	256	OS
GEO: GSE31684	Affymetrix Human Genome U133 Plus 2.0 Array	93	OS
GEO: GSE32548	Illumina HumanHT-12 V3.0 expression beadchip	131	OS
GEO: GSE48075	Illumina HumanHT-12 V3.0 expression beadchip	142	OS
GEO: GSE48276	Illumina HumanHT-12 WG-DASL V4.0 R2 expression beadchip	116	OS
GEO: GSE70691	Illumina HumanHT-12 WG-DASL V4.0 R2 expression beadchip	49	OS
ArrayExpress: E-MTAB-4321	Illumina HiSeq 2000	476	PFS
IMvigor210	Illumina RNAseq	348	OS

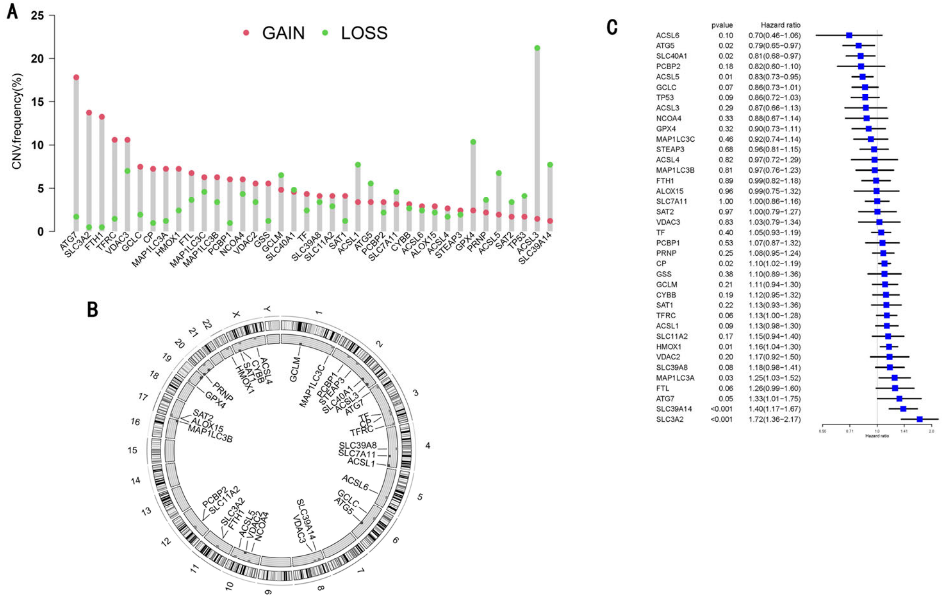

Fig. S1. Prognostic analysis and mutation relevance of 38 ferroptosis-related genes. (A) The frequency of CNV variation of ferroptosis-related genes in a TCGA dataset. The frequency of alteration was indicated by the column height, green dot indicated frequency of deletion and red dot indicated frequency of amplification. (B) The CNV alteration location of ferroptosis-related genes on 23 chromosomes by TCGA dataset. (C) The mutation relevance of 38 genes relevant to ferroptosis (co-occurrence was shown in dark green and mutually exclusive was shown in brown). (B) The prognostic analysis performed with univariate Cox regression model in 38 ferroptosis-related genes in the six bladder cancer datasets. Factors with Hazard ratio >1 was risky and factors with hazard ratio <1 were protective..

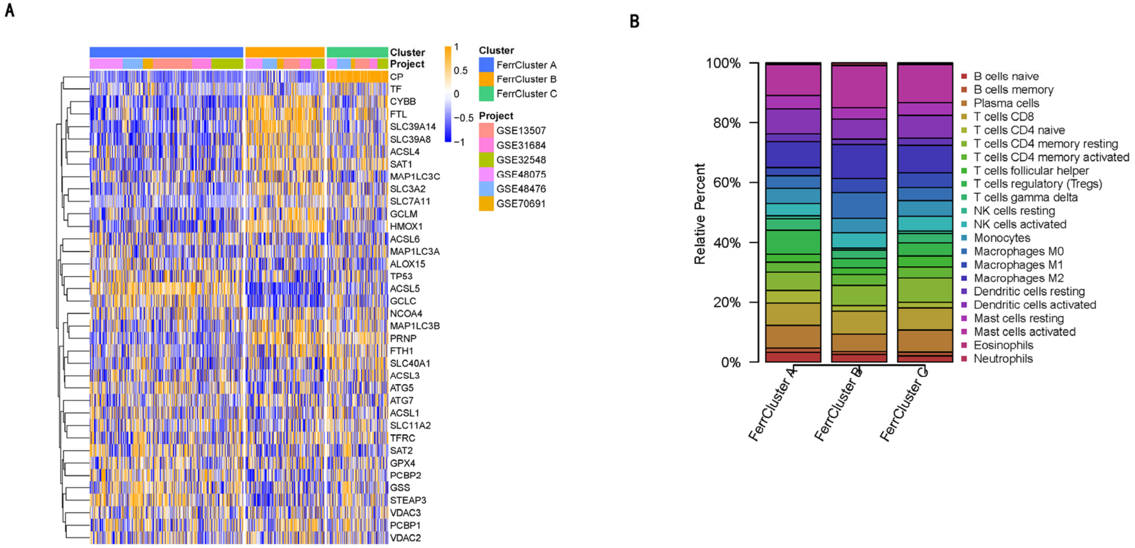

Fig. S2. Non-monitored clustering of 38 ferroptosis-related genes. (A) Non-monitored clustering of 38 ferroptosis-related genes in the six bladder cancer datasets. The ferroptosis clusters and dataset names were used as patient annotations. Each column represented patients and each row represented ferroptosis-related genes. (B) The composition diversities of infiltrating immune cell in three ferroptosis forms analyzed by CIBERSORT tool.

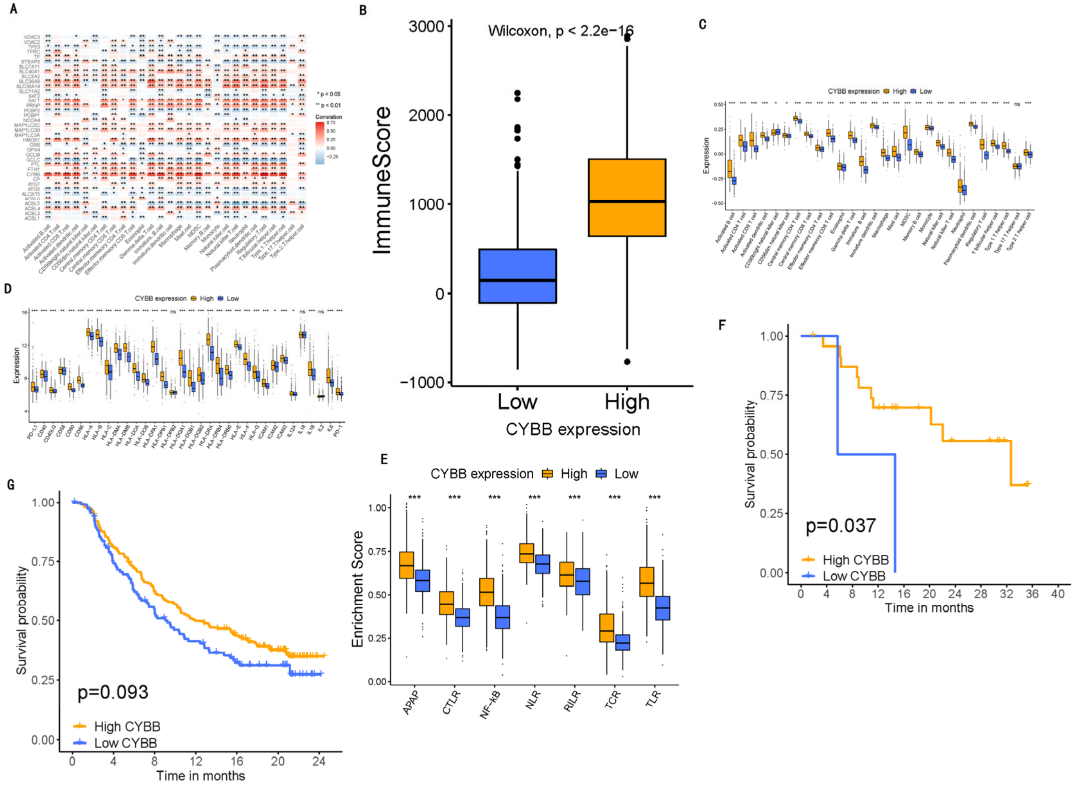

Fig. S3. Ferroptosis-related genes were correlated with TME infiltrating cells and CYBB played an role in action of TME infiltrating cells. (A) Spearman analyses was performed to reveal the correlation between different types of TME infiltration cells and ferroptosis-related genes. It shows negative correlation in the color of blue and positive correlation in the color of red. (*P less than 0.05; **P less than 0.01) (B) Diversity in immuneScore between high and low CYBB expression. (P less than 0.05, Wilcoxon test) (C) Diversity in the abundance of each TME infiltrating cell between CYBB high and low expression groups. (D) Variation in abundance of MHC molecules, costimulatory molecules, interleukin, and adhesion molecule between CYBB high and low expression groups. The interquartile value scale was indicated by boxes' two ends. The median value was indicated by those lines in the boxes, and outliers were shown with dots of black color. The value of P was graphically shown by asterisks (*:P less than 0.05; **:P less than 0.01; ***:P less than 0.001). (E) Diversities in immune-activated pathways between CYBB high and low expression groups. APAP, antigen processing and presentation; CTLR, type-C lectin receptors signaling pathway; NLR, NOD-like receptors; TCR, thymus dependent lymphocyte receptors; TLR, Toll-like receptors, RILR, RIG-I-like receptor signaling pathway (***P less than 0.001) (F,G) Survival analysis performed with Kaplan-Meier curves in patients with low or high CYBB transcripts in the anti-PD-L1 (F) and anti-PD-1 (G) immunotherapy datasets.

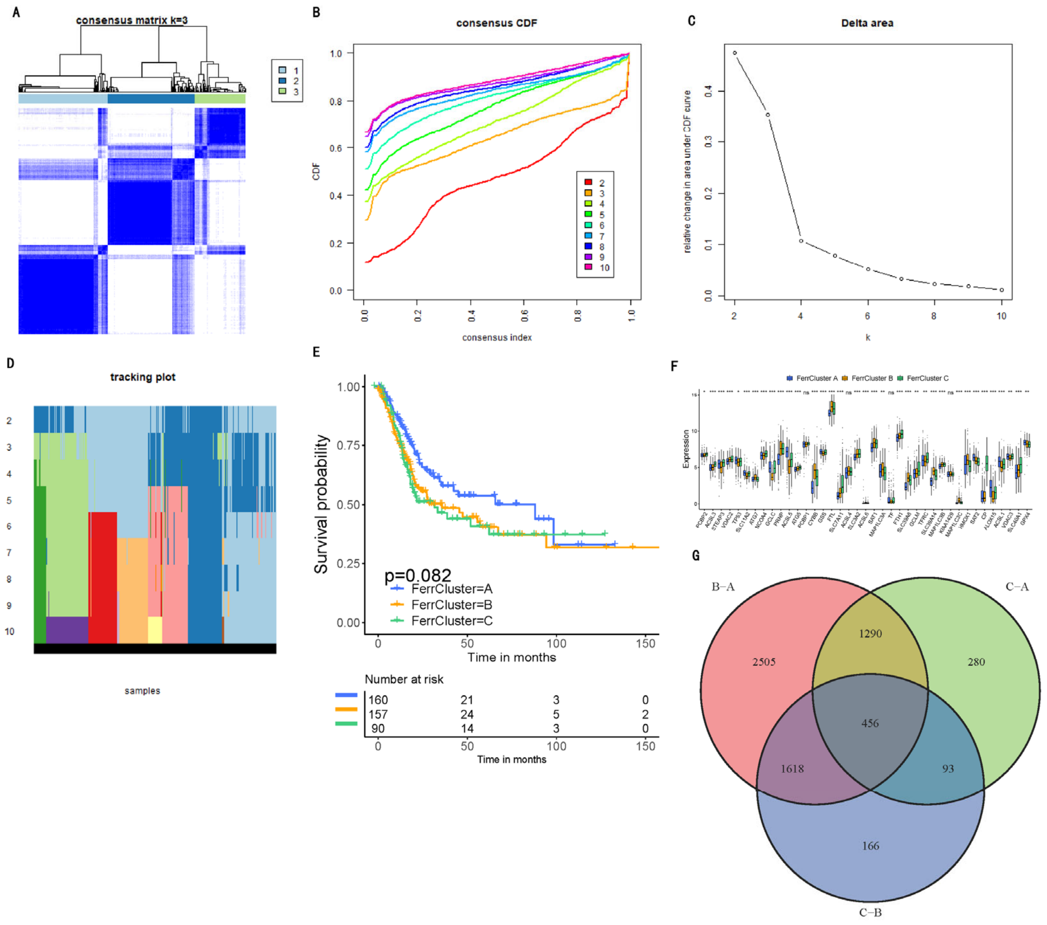

Fig. S4. Non-monitored clustering of 38 ferroptosis-related genes in the TCGA: BLCA dataset. (A-D) Consensus matrices of the TCGA: BLCA dataset, k = 3. (E) Survival analysis performed with Kaplan-Meier curves for ferroptosis cluster-A (160 patients), ferroptosis cluster-B (157 patients) and ferroptosis cluster-C (90 patients) in TCGA: BLCA dataset. Overall, the ferroptosis cluster B suggested better survival outcome than the other two ferroptosis clusters.(p=0.082, Log-rank test) (F) The transcript level of 38 ferroptosis-related genes in the three ferroptosis clusters. The interquartile value scale was indicated by boxes' two ends. The median value was indicated by those lines in the boxes, and outliers were shown with dots of black color. The value of P was graphically shown by asterisks (*:P less than 0.05; **:P less than 0.01; ***:P less than 0.001). (G) 456 ferroptosis pattern-related genes shown in venn diagram.

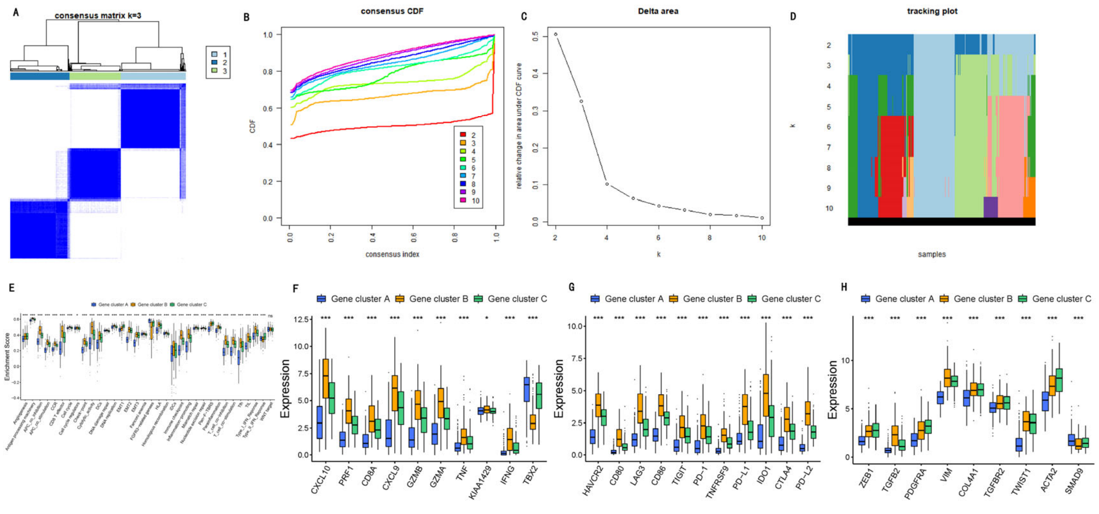

Fig. S5. Transcriptional changes of cytokine, chemokine and accepted features in different gene clusters. (A-D) Non-monitored clustering of 456 ferroptosis subtype-related genes in TCGA: BLCA dataset and consensus matrices for k=3. (E) Diversity in the abundance of accepted features including stromal-activation related signatures, tumor-promotion related signatures and immune-activation related signatures among these gene clusters. (F) Transcriptional diversity of genes relevant to immune-activation in these gene clusters. (G) Transcriptional diversity of genes relevant to immune-checkpoint in these gene clusters. (H) Transcriptional diversity of genes relevant to TGFβ-EMT pathway in these gene clusters. The interquartile value scale was indicated by boxes' two ends. The median value was indicated by those lines in the boxes, and outliers were shown with dots of black color. The value of P was graphically shown by asterisks (*:P less than 0.05; **:P less than 0.01; ***:P less than 0.001).

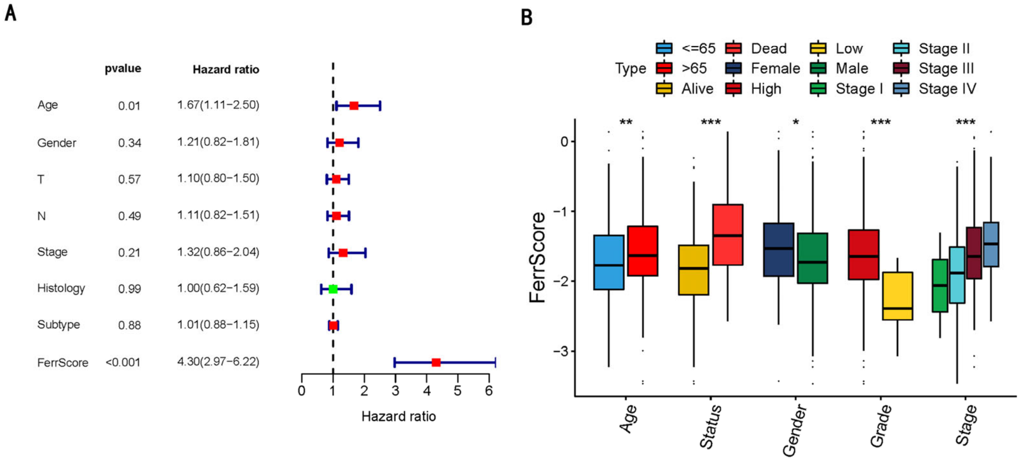

Fig. S6. The value of FerrScore in prognosis and its relevance to the clinicopathologic characters. (A) Multivariate Cox regression analysis for FerrScore in TCGA: BLCA cohort shown by the forest plot. (B) Difference in FerrScore among distinct clinical subgroups in TCGA: BLCA cohort. (*:P less than 0.05; **:P less than 0.01; ***:P less than 0.001)

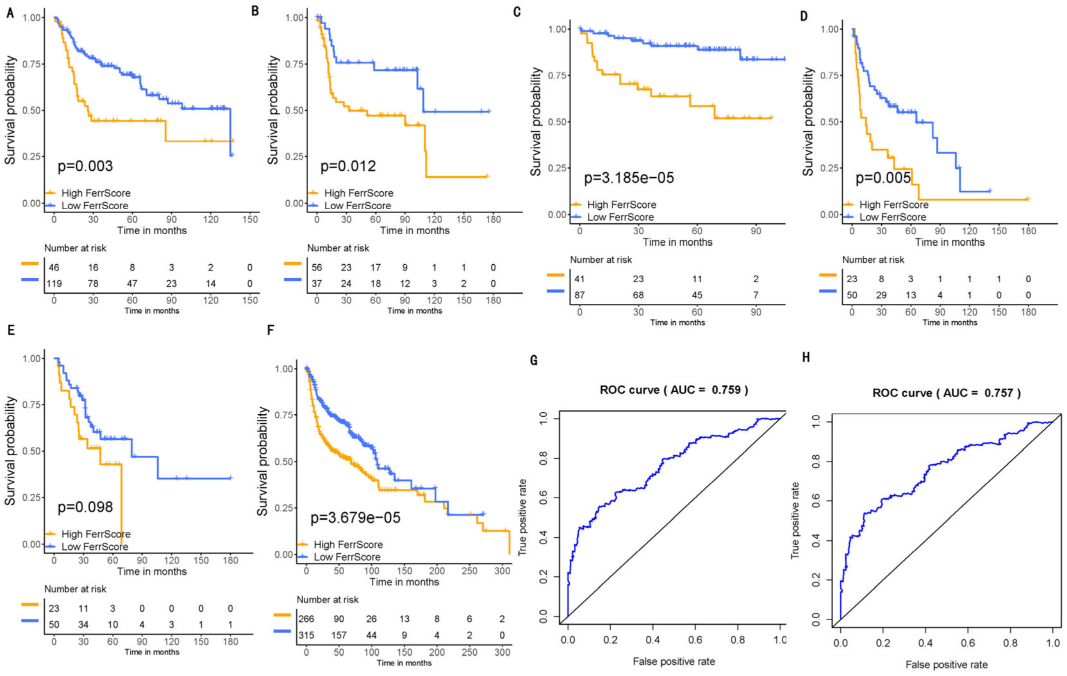

Fig. S7. Prognostic value of FerrScore in other independent BLCA cohorts. (A) GSE13507: $p < 0.05$, Log-rank test. (B) GSE31684: $p < 0.05$, Log-rank test. (C) GSE32548: $p < 0.05$, Log-rank test. (D) GSE48075: $p < 0.05$, Log-rank test. (E) GSE48276: $p < 0.05$, Log-rank test. (F) All GEO bladder cancer cohorts: $p<0.05$, Log-rank test. (G) The prognostic value of FerrScore in bladder cancer cohorts for three year survival. AUC=0.759. (H) The prognostic value of FerrScore in bladder cancer cohorts for five year survival. AUC=0.757.

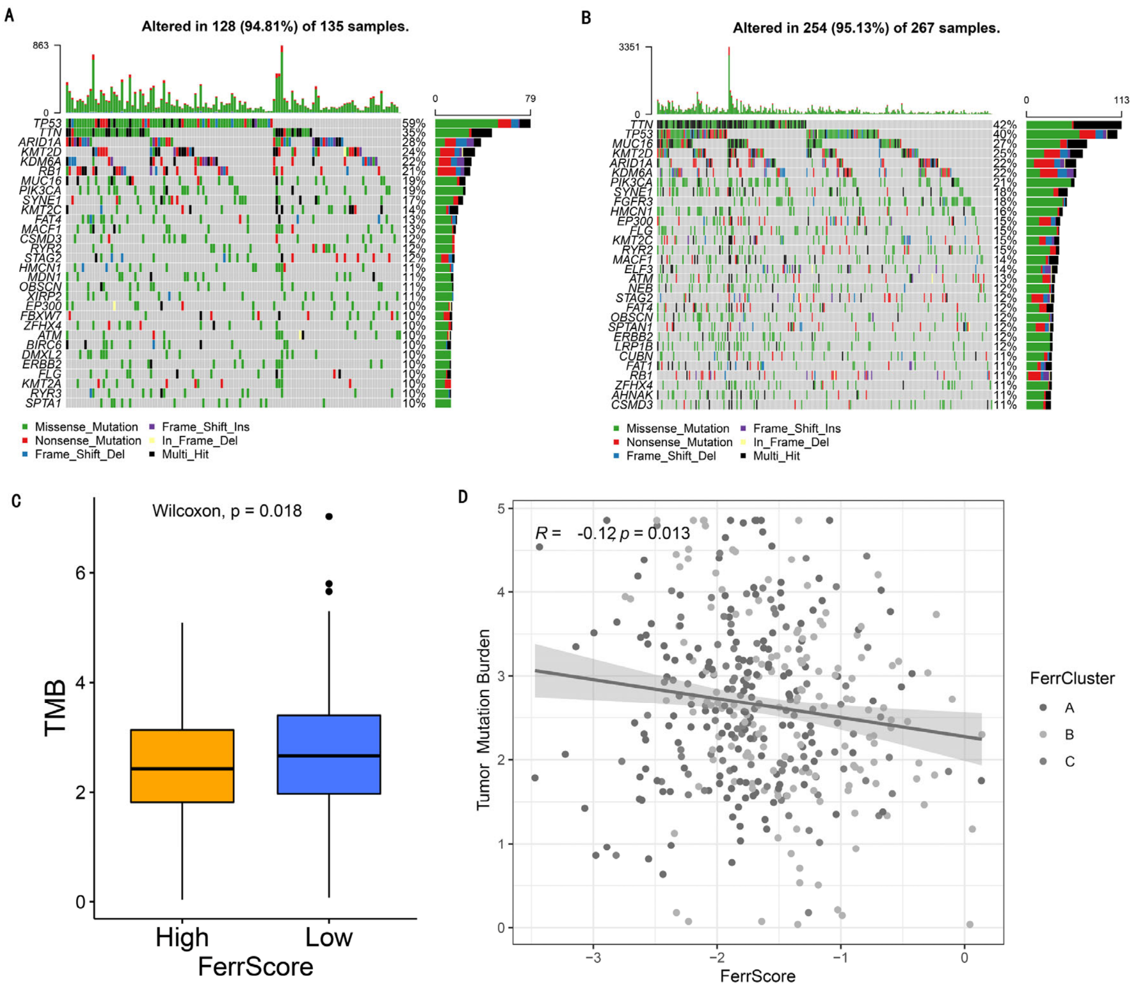

Fig. S8. Characteristics of ferroptosis in tumor somatic mutation.Somatic mutation between low and high FerrScore in TCGA-BLCA dataset was analyzed using maftools package. The waterfall plot of tumor somatic mutation in patients with high FerrScore (A) and low FerrScore (B). each patients were shown individually in separate columes. TMB was shown on the upper bar graph, and the frequency of each gene mutation was indicated by the number on the right. Each variant type's occupation was shown on the right bar graph. (C)The TMB in the high and low FerrScore groups. (Wilcoxon test, p=0.018) (D) The correlation between FerrScore and TMB (*:P less than 0.05; **:P less than 0.01; ***:P less than 0.001). TMB means tumor mutation burden.
